# Using the International Alcohol Control (IAC) policy index to assess effects of legislative change in Aotearoa New Zealand

**DOI:** 10.1186/s12889-024-18992-y

**Published:** 2024-06-11

**Authors:** Sally Casswell, Steve Randerson, Karl Parker, Taisia Huckle

**Affiliations:** SHORE & Whariki Research Centre, College of Health, Massey University, PO Box 6137, Victoria Street West, Auckland, 1142 New Zealand

**Keywords:** Alcohol policy, IAC Policy Index, Stringency, Impact, Framework

## Abstract

**Background:**

The IAC Policy Index was developed to allow comparison in alcohol policy between countries and within countries over time including in low resource settings. It measures four effective alcohol policies and takes into account stringency of regulation and the actual impact on the alcohol environment, such as trading hours and prices paid. This framework was used to assess policy in Aotearoa New Zealand in a time period covering two relevant legislative changes. This is the first study to use an alcohol policy index to assess and describe legislative change within country.

**Methods:**

Data to calculate the IAC Policy Index was collected for 2013 and 2022. Stringency of policy was assessed from legislative statutes and impacts of policy on the alcohol environment from administrative data and specifically designed data collection.

**Results:**

The overall IAC Policy Index score improved over the time period. The scores for the separate policy areas reflected the legislative changes as hypothesised, but also independent changes in impact, given ecological changes including reduced enforcement of drink driving countermeasures and increased exposure to marketing in digital channels. The IAC Policy index reflects the changes in policy status observed in Aotearoa, NZ.

**Discussion:**

The IAC Policy Index provided a useful framework to assess and describe change in alcohol legislation contextualised by other influences on policy impact over time within a country. The results indicated the value of assessing stringency and impact separately as these moved independently.

**Conclusions:**

The IAC Alcohol Policy Index, measuring both stringency and actual impact on the alcohol environment with a focus on only the most effective alcohol policies provides meaningful insights into within-country policy strength over time. The IAC Policy Index used over time can communicate to policy makers successes and gaps in alcohol policy.

## Background

### The International Alcohol Control (IAC) policy index

The IAC Policy Index is a development of the International Alcohol Control (IAC) study which originated in Aotearoa, New Zealand (NZ) in 2011 and has engaged collaboratively with 34 countries over a period of 12 years [1]. The IAC Policy Index (Table 1) was developed using data from high and middle income countries [2]. It was strongly associated with recorded consumption of alcohol per capita (APC) in diverse country settings and showed a larger relationship with APC than previously published indices [2]. The IAC Policy Index has also been positively correlated with levels of abstention across countries and alcohol consumption [3]; the findings showed that people who lived in countries with more stringent alcohol policies drank less than people in countries with less strict policies. The more stringent policies were associated with reduced drinking overall and showed more significant associations in young adult drinkers and those with fewer years of education [4].

**Table 1:**
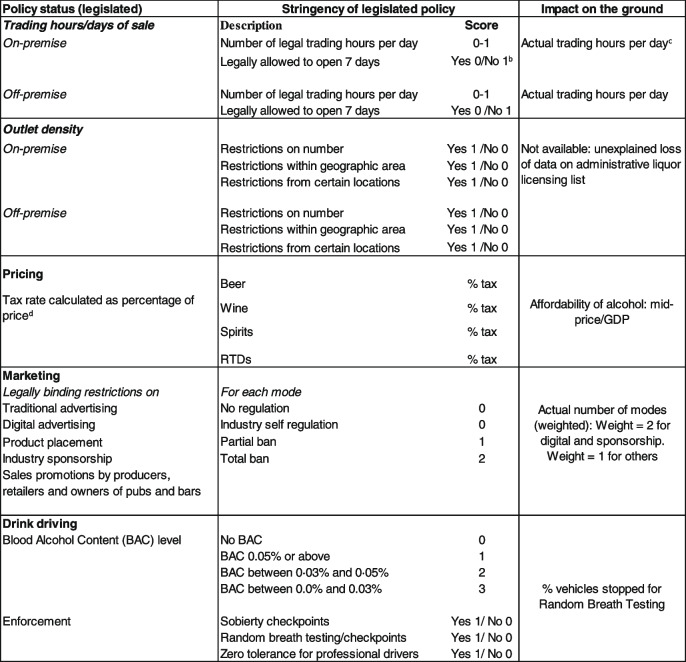
Measures used in the IAC Policy Index^a^

^a^Additions were made to this index, compared to an index we previously published [2], to include data available for Aotearoa, New Zealand e.g., inclusion of RTDs in pricing to reflect our market, and the addition of weights to reflect environmental conditions of increased digital marketing (impact on the ground)

^b^Yes/No were scored depending on which option represented the restriction, e.g., if no meant greater restriction then it was coded as 1

^c^Same scoring as used for legal hours

^d^Weighted by the % of beverages consumed in NZ as per WHO: Global Information System on Alcohol and Health (GISAH)

The IAC Policy Index differs from previous alcohol policy indices by intentionally focusing on a small number of highly effective policies supported by extensive research [5, 6]. These policies target reducing availability, affordability, and marketing of alcohol, with drink-driving countermeasures also included despite their higher costs. Additionally, the index introduces an innovative approach by incorporating not only the strictness of legislation but also measures reflecting the real-world impact of these policies, such as actual alcohol availability hours, affordability, and enforcement of drink-driving laws [2]. Unlike previous indices, which mainly assess regulatory stringency (e.g., [7]) and sometimes enforcement (e.g., [8]), the IAC Policy Index evaluates both regulatory stringency and the broader policy impact on the ground.

The design of the IAC Policy Index was intended to provide a metric to allow comparison of the strength of alcohol policy between countries and also a framework to allow assessing policy change within country over time.

### Alcohol policy changes in Aotearoa,^1^ New Zealand

The Sale and Supply of Alcohol Act (SSAA) 2012 was reviewed primarily due to concerns surrounding high rates of alcohol-related harm and its societal impact [9]. The SSAA made some key changes relevant to reduction of alcohol availability. Specifically, these included a reduction in maximum trading hours from 24 h of legal opening to mandatory closing of 11 pm off premise and 4.00am for on premises. Also included were new non-mandatory Local Alcohol Policies (LAPs) which were intended to give local authorities the ability to address the location, number and trading hours of alcohol outlets (if local authorities opted for implementation).

The response to marketing was largely unchanged, with reliance on a voluntary code. However, new partial restrictions were introduced in the SSAA covering advertising with special appeal to minors, certain special offers and price discounts over 25% (if visible outside licensed premises) and the prohibition of marketing which ‘encourages people, or is likely to encourage people, to consume alcohol to an excessive extent’ was broadened from applying only to licensed premises to state: ‘whether on licensed premises or at any other place’.

Another relevant legislative change was an amendment in 2014 to the Land Transport Act 1998 which lowered the adult limit from 400mcg of alcohol per litre of breath to 250mcg of alcohol per litre of breath (from 80 mg of alcohol per 100 ml of blood to 50 mg of alcohol per 100 ml of blood) [10].

During this period there were no policy changes affecting pricing policy. The existing policy allowed for excise tax on alcohol to be adjusted in line with inflation throughout this period and inflation averaged 2.4% per annum over this time.

The aims of this study were to provide a descriptive assessment of policy status including the ‘impact on the ground’ of policy over time in Aotearoa, NZ. A secondary aim was to assess if the IAC Policy index scores reflected the changes in policy status and impact on the ground observed in Aotearoa, NZ. Given the nature of the legislative changes it was hypothesised there would be improvement in scores in the availability and drink driving domains but no changes in other domains.

This is the first analysis using the IAC Policy Index, and among the first alcohol policy indices, to assess change in stringency and impact of policies following relevant legislative change over time within a country.

## Method

The design of the study was descriptive. The IAC Alcohol Policy Tool was used to collect data in 2013 and 2022 and these data were used to calculate the IAC Policy Index in both years.

### Data sources and measures

The Alcohol Environment Protocol), the precursor of the online IAC Alcohol Policy Tool was developed in a cross country collaborative project to allow countries to document and assess (in a comparable way) the policy environment in which alcohol is sold and consumed [11].

This was utilised in Aotearoa, NZ in 2013 prior to the key policy changes of the SSAA being implemented in December 2013 and 2022. By 2022 the tool had been streamlined and transferred to an online tool, the IAC Alcohol Policy Tool (APT).

The APT data records whether key policies are in place, their stringency (i.e., the level of restriction), and ‘policy impact on the ground’ measures of the alcohol environment in relation to each domain. For a description of the measures included in each policy domain see Table 1. It does not include outcome measures such as consumption and harm data. Before analysis the data were cross-checked among the research team.

### Stringency

Data on policies and their stringency were drawn from legislative statutes and administrative documents. For physical availability two measures were included: laws and regulations about alcohol outlet density and hours of trading. Data was collected on legal restrictions, if any, on number; specified geographic area; distance from certain locations for outlets; and number of hours stores were permitted to be open per day and if premises were permitted to be open for all 7 days of the week. As some areas had shorter legal trading hours due to the implementation of LAPs an average of hours was used, weighted by population.

The drink driving policy stringency scale ranged from not having a BAC limit through to a BAC between 0%—0·03% (up to 400 mcg) (the strictest). Data were also collected about whether sobriety checkpoints (where suspicion of drinking is required before testing can occur), random breath testing checkpoints (where any driver can be tested without suspicion of drinking), and/or random breath testing (where any driver can be stopped anywhere and tested) were implemented.

For tax, policy stringency was based on the share of the retail price comprised of tax based on the tax rates. The tax was calculated as a percentage of retail price for four beverages (beer, wine, spirits, and RTDs) weighted by the proportion each beverage contributed to the alcohol market in NZ.

The marketing domain was made up of five categories – traditional advertising (e.g., TV, radio, print, mail, point of sale merchandise), digital advertising (e.g., social media, online, apps), product placement (e.g., movies, TV), sponsorship (e.g., sports, music, educational programs, charities), and sales incentives (e.g., price, competitions, merchandise). Scores available were: no regulation/industry voluntary code; a partial ban and a total ban, with total ban receiving the highest score.

### Policy impact

Data for the impact measures were collected as follows:

Actual days of sale and trading hours: a random sample of 200 outlets was drawn from the national list of licenced premises in Aotearoa, NZ available from the Ministry of Justice website [12]. Prior to random selection, we refined this sampling list to include bars/nightclubs, restaurants, sports clubs, alcohol shops and supermarkets as these reflected the most common places where alcohol is consumed or purchased in Aotearoa, NZ. To obtain actual days of sale and trading hours, we collected data from a minimum of 20 on-premises outlets and 20 off-premises outlets and asked about opening hours on each day in a typical week and days of opening. For analysis, the trading hours were an average of actual hours of trading across all days for on and off-premises.

Density of alcohol outlets: data were unable to be obtained due to the administrative data on actual number of outlets in Aotearoa, NZ not being comparable between 2013 and 2022 due to an unexplained loss of data.

Affordability of alcohol: the affordability of alcohol was assessed to gauge the impact of tax policy. Surveys of premises were undertaken by phoning a random sample of common types of on- and off-premises to document retail prices. Outlets were called until saturation was reached—defined as the point where any new prices gathered fell within $1.50 of the range of prices already gathered for on-premises and off-premises separately (data for prices was supplemented with searching online for off-premises prices). The typical mid-price of 15 ml absolute alcohol was averaged over the four most common commercial beverages, beer, wine spirits and ready to drinks, weighted by the proportion each beverage contributed to the alcohol market (as defined by WHO data) [2, 13], and then divided by per capita GDP to create a measure of affordability.

For drink driving countermeasures, the percentage of vehicles on the road stopped for random breath testing, available from Police data, was used to assess the impact of the policy. The denominator included all vehicles in Aotearoa, NZ excluding trailers.

Alcohol marketing: the policy researchers completed a schedule documenting the modes in which alcohol marketing was present or absent, including traditional advertising, sponsorship and digital including social media. The schedule comprised a list of 31 items (yes/no) to cover the full range of marketing activities. Digital and sponsorship were given a higher weight (2) than general (1) to reflect the relative power that these methods likely have [14, 15]. In 2022 additional modes within the digital channel were added, reflecting the expansion of marketing in the digital media.

### The IAC policy index – analysis

The IAC Policy Index generated scores with a potential range of 0 to 25 points. In each domain, data collected for the Index were converted into a score between zero and one, with a higher score representing more stringent policy and evidence of more restrictive on-the-ground impact. Some of the data collected had to be inversed so that this direction was maintained. Once standardised, values in each domain were then weighted by between one and five to reflect effectiveness based on the available scientific evidence [6] and then summed to make up the total IAC Policy Index (out of 25) [2]. The sensitivity analysis was then undertaken to assess the robustness of the Index to changes in the effectiveness weights applied in the Index domains and to select the final weights for the Index (this sensitivity analysis was undertaken as part of a previous study). Further details of the measures, effectiveness weighting and sensitivity analysis of the weights and calculation of the Index are available in Casswell et al. [2].

## Results

The IAC Policy Index score increased overall from 2013 to 2022, indicating a small improvement in alcohol policy in Aotearoa, NZ (2.4%), however there was considerable variation between domains (Table 2). The domain which showed the most improvement was hours and days of sale (165.9%).

**Table 2:** IAC Policy Index scores in Aotearoa, NZ before and after SSAA implementation (maximum score available in each year is 25)

	2013	2022
Hours and days of sale (Max score = 2)	0.41	1.09
Outlet density (Max score = 3)	0.00	0.00
Drink driving (Max score = 4)	2.37	2.35
Pricing (Max score = 9)	1.30	1.27
Marketing (Max score = 7)	1.70	1.21
Total (Max score = 25)	5.78	5.92

**Fig. 1:**
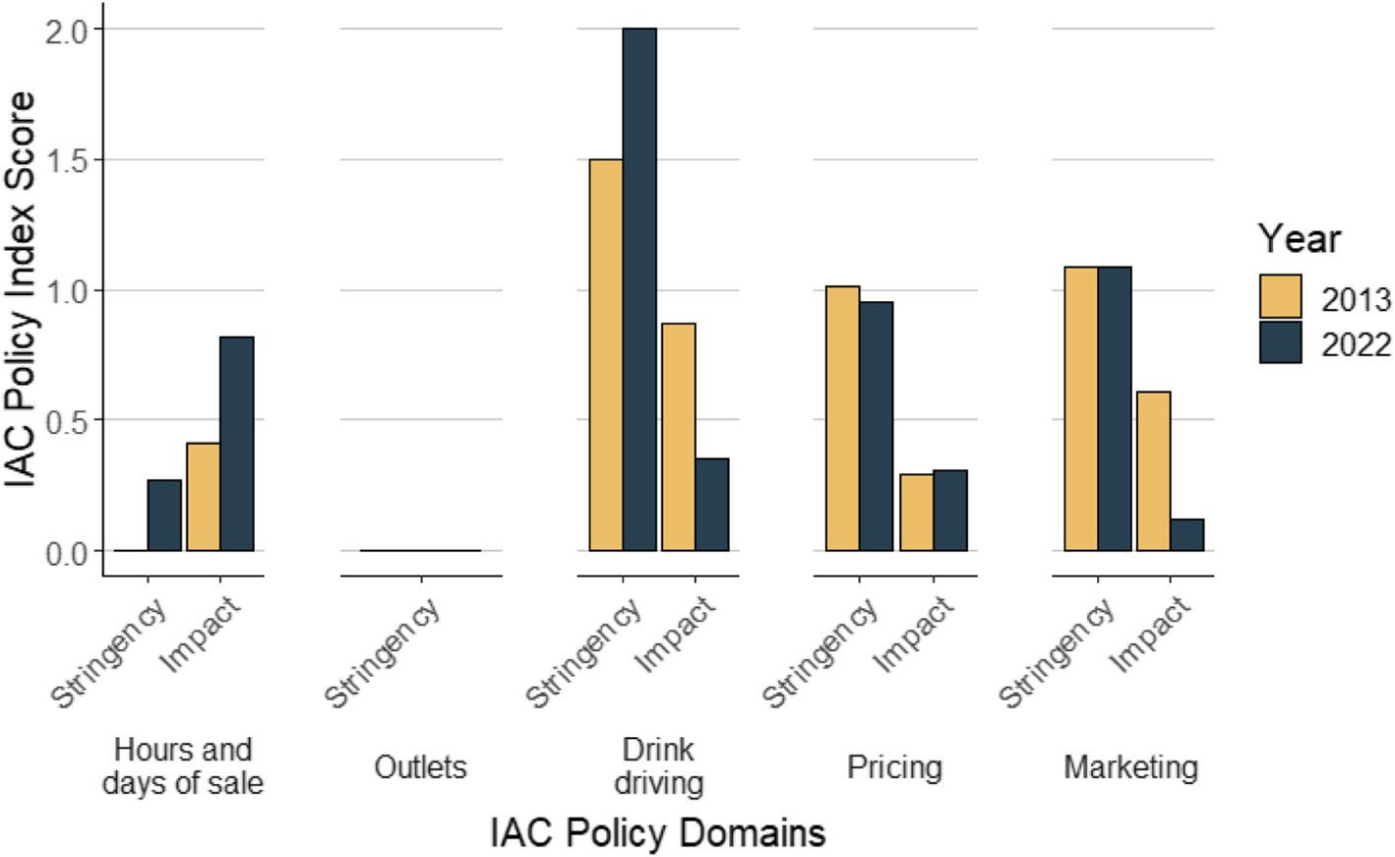
Stringency and Impact for each domain compared by year*. *The bars have no height when the score is zero

There was also variation between the extent to which the stringency score and the impact score contributed to the overall score for each domain (Fig. 1).

Changes in the domain scores indicated increased stringency of policy in trading hours (assessed as zero in 2013 as 24-h trading was legally possible) and this was also reflected in policy impact. Outlet density reflected no change in stringency due to no legislative change, and data on impact was not available. Drink driving countermeasures were shown to increase in stringency but reduce in policy impact. Pricing was relatively unchanged and marketing showed a decrease in policy impact.

## Discussion

The changes in the IAC Policy Index scores in relation to trading hours and drink driving legislation are as hypothesised given the changes in legislation in Aotearoa, NZ. However, the separate assessment of stringency and impact provided additional information about the policy environment.

In the case of trading hours there is an improved score in both stringency and impact reflecting the law change restricting 24 h opening and that it translated into actual hours of opening. The LAPs that were implemented reduced trading hours (on average about two hours on premise and one hour off premise), however, less than 35% of our population resides in an area where an LAP is in force [16]. (LAPs were held up, including in some of our largest cities, by appeals which have been overwhelmingly made by alcohol industry actors. [17]) The IAC Policy Index was able to detect these changes in policy status and impact on the ground. A significant reduction in late night assaults following enactment of the SSAA was found for on premise outlets from Police data and was also reflected in hospitalisations [18, 19].

The greater stringency introduced in drink-driving policy, by contrast, did not translate to policy impact on the ground included in the IAC Policy Index as there was a substantial reduction in Police implementation of random breath testing from 2.9 million tests per year in 2013 to 1.6 million tests in 2022. This lower volume of enforcement has on average been sustained since 2015 [20]. Contextual information shows that alcohol-related crashes involving death or serious injury increased, particularly between 2014 (following the introduction of the reduced BAC) until 2017 [21].

Marketing continued to be largely unregulated, and the partial regulations introduced in the 2012 SSAA have resulted mostly in education of licensees and two cases of prosecution before the Alcohol Regulatory and Licensing Authority (Pers. comm. Rob Abbott, Alcohol Licensing Inspector & Principal Specialist, Alcohol Licensing & Environmental Health Licensing & Regulatory Compliance, Auckland Council). Relatively low levels of legal enforcement may have reflected a perception of difficulty of interpretation of the legislation (pers. comm. Inspector Hamish Milne, Manager: Alcohol Harm Prevention Māori, Community, Prevention and Partnerships, NZ Police).

The impact of alcohol marketing on the ground has increased because of the advent of digital marketing, which has increased the power of the marketing of alcohol products [14, 22].

Similarly alcohol taxation policy has not changed in Aotearoa, NZ but, despite annual adjustment for inflation, affordability of alcohol products has increased [23].

Although data on outlets was not included in the IAC Policy Index (due to the loss of data in 2014), this domain is unlikely to have made any difference to Aotearoa, NZ’s policy strength. SSAA did not introduce binding regulation of outlet location or density and the data on licenses available since 2015 shows no meaningful change; the total number of licences decreased 2.9% from 2015 to 2022.

Over this time period in which alcohol policy strength as measured by the overall IAC Policy Index increased by 2.4% the alcohol available for consumption, which provides a measure of average per capita consumption, decreased from 9.23 L in 2013 to 8.66 L in 2022 [24].

Interpreting scores from the IAC Policy Index requires several considerations. While the overall score provides a summary score of integrated policies, individual domain scores may show larger improvements in response to policy adjustments, as shown in this study for days and hours of sale, however, other domains may score in the opposite direction, influencing the overall score differently. Thus, both domain-specific and overall scores are useful for interpreting the index over time. While other research data can contextualise findings, it is not essential for understanding changes in policy status in a country over time. The IAC Policy Index collects its own impact on the ground measures for this purpose and bases its rationale on extensive research evidence of effective policies.

A strength of the current analysis is the inclusion of data on measures of impact on the ground which incorporates compliance, something not often included in indices of policy strength; the current analysis also covered a span of a decade which allowed for the uptake and implementation of policies [25].

### Limitations

Lack of data is a limitation. The lack of impact measures for outlet density and spatial positioning needs to be considered when interpreting results, although the overall change in outlet density in recent years has been minimal so this may have little effect on the results.

To collect data on actual trading hours, we called 21 off -premises and 36 on-premises. While our sample was random, it was relatively small and this should be taken into account when considering the findings.

## Conclusions

The IAC Policy Index provided a useful framework to assess, summarise and communicate change in alcohol policy over time within country based on four effective policies. The data collected showed different trends in stringency and impact. Impact in some domains was reduced even in the absence of any change in stringency, reflecting ecological changes such as increased exposure to digital marketing and reduced police enforcement. Measuring both stringency and impact are useful elements of a meaningful alcohol policy index.

## Data Availability

The study data are available at: https://www.kaggle.com/datasets/karlparker/nz-iac-policy-index-data.

## Footnotes

1 Aotearoa is the Māori (indigenous) name for New Zealand.
